# Hydrogen bonding patterns in the cocrystals of 5-nitrouracil with several donor and acceptor molecules

**DOI:** 10.1186/1860-5397-1-15

**Published:** 2005-12-09

**Authors:** Reji Thomas, R Srinivasa Gopalan, G U Kulkarni, C N R Rao

**Affiliations:** 1 Chemistry and Physics of Materials Unit, Jawaharlal Nehru Centre for Advanced Scientific Research, Jakkur P.O., Bangalore 560 064, India

## Abstract

Cocrystals of 5-nitrouracil with solvent molecules, dioxane, pyridine, DMSO, formamide and ethanol as well as with piperazine, N, N'-dimethylpiperazine, 3-aminopyridine and diazabicyclo [2.2.2]octane obtained by deliberate inclusion, have been examined by X-ray crystallography. The tape structure found in the parent centric form of nitrouracil is retained with some modifications in the cocrystals with dioxane, piperazine, diazabicyclo [2.2.2]octane, N,N'-dimethylpiperazine, pyridine and DMSO, with the guest molecules forming alternate tapes. In cocrystals involving formamide, ethanol and 3-aminopyridine, the molecular tapes exhibit mixed compositions. The observed bonding patterns have been classified into six schemes. Interestingly, quadruple type hydrogen bonding patterns are seen in cocrystals containing 3-aminopyridine or ethanol and water, while a network of acyclic tetrahedral pentamers of water is found in the cocrystal containing diazabicyclo [2.2.2]octane and water.

## Introduction

Crystalline molecular solids exhibit properties- chemical reactivity, electrical, optical or magnetic – unique to their crystal structure. This justifies the growing interest in designing and preparing molecular crystals with desired properties and understanding crystal packing, which are clearly the mandates of the field of crystal engineering. [1–5] Hydrogen bond is almost universally present in molecular crystals. The work in this field is therefore centered on rationalizing and predicting hydrogen bonding patterns and motifs involved in the crystal structures. [6–11] In this context, influence of the solvent media on the crystallization process assumes significance. Molecules tend to crystallize in different polymorphic structures, depending on the nature of the solvent and the crystallization conditions employed,[12] an archetypal example being *trans*-cinnamic acid which crystallizes in three polymorphic forms, of which only two can undergo photochemical dimerizaton. [13–15] Another possibility is inclusion of the solvent molecules in the crystal structure of the solvate forming a cocrystal. [16–18] For example, when p,p'-biphenol is crystallized from DMSO, two forms of cocrystals are formed, one with a tunnel structure and the other having a layered structure. [17] Cocrystallization of desired molecules other than the solvent can also be induced under suitable conditions. [19–20] An interesting example comes from the work of Ma and Coppens,[19] who reported the formation of pyrene nanorods within a supramolecular framework.

5-nitrouracil, **I**, is an interesting molecule which can be crystallized in centric and non-centric structures. [21–22] The centric structure obtained by crystallizing **I** from water, exhibits tapes of nitrouracil molecules, each of which forms on either side, linear N-H...O hydrogen bonds in cyclic R_2_^2^(8) arrangement (H...O distance, 1.81–1.86 Å). It also forms C-H...O linear bonds with a neighboring tape engaging oxygen of the nitro group. In the present study, we have investigated how the hydrogen-bonded structure of **I** is affected when it is cocrystallized with different molecules. For this purpose, we have crystallized **I** from different solvents such as dioxane, pyridine, dimethylsulfoxide (DMSO), formamide and aqueous ethanol, of which the first three are clearly electron donors. In addition, we have obtained cocrystals of **I** with other electron donor compounds such as piperazine, N,N'-dimethylpiperazine, 3-aminopyridine and diazabicyclo [2.2.2]octane. Crystallographic structures of the cocrystals so obtained have revealed interesting changes in the hydrogen bonding pattern of **I** in the presence of other molecules, strong electron donors destroying completely the dimeric N-H...O bonds characteristic of the parent system of **I**.

## Experimental

All chemicals were used as purchased from Aldrich without further purification. Cocrystals of **I** with DMSO, formamide, pyridine, dioxane and ethanol were obtained by slow evaporation of a solution of **I** in respective solvents. Cocrystals of **I** with non-solvent guests – piperazine, N,N'-dimethylpiperazine, 3-aminopyridine and diazabicyclo [2.2.2]octane (DABCO)- were made by solvothermal method. In all the cases, the compound **I** and the guest molecule were taken in 1:1 ratio in aqueous methanol and stirred for 1 h at ambient temperature. The final suspension was placed inside a Teflon-lined autoclave (21 cm^3^, 70% filling). The reaction was carried out at 145°C under autogenous pressure for 24 h. The autoclave was removed and left at ambient temperature for 12 h before opening. The crystals obtained were examined under an optical microscope and those suitable for single-crystal diffraction were separated out.

X-ray diffraction intensities were measured at room temperature (298 K) by ω scans using a Siemens three-circle diffractometer attached to a CCD area detector and a graphite monochromator for the Mo-Kα radiation (50 kV, 40 mA). Initially, the unit cell parameters and the orientation matrix of the crystal were determined using ~45 reflections from 25 frames collected over a small ω scan of 7.5°, sliced at 0.3° interval. A hemisphere of reciprocal space was then collected using the SMART software[23] with the 2θ setting of the detector at 28°. The data reduction was performed using SAINT program[23] and the orientation matrix along with the detector and the cell parameters were refined for every 40 frames on all the measured reflections. The crystal structures were solved by direct methods using the SHELXTL program[24] and refined by full matrix least squares on F^2^ (CCDC 267375 – 267382 and 266627). All non-hydrogen atoms were refined anisotropically. Hydrogen atoms were located by the difference Fourier synthesis and refined isotropically and were then normalized to the average neutron diffraction values. [25] Hydrogen bond analysis was carried out using the PLATON routine. [26] (see supplimentary information). Crystal structure data of the cocrystals are given in Table 1. All except with DMSO[21] are being reported for the first time. From Table 1, we observe that cocrystals from all non-solvent guests differ from the initial ratio of 1:1.

**Table 1:** Crystal structure data of cocrystals

	1	2	3	4	5	6	7	8

Empirical formula	C_4_H_3_N_3_O_4_. C_2_H_4_O_1_	C_4_H_3_N_3_O_4_. 2C_2_H_5_N_1_	C_4_H_3_N_3_O_4_. C_3_H_7_N_1_	C_4_H_3_N_3_O_4_. C_5_H_5_N_1_	2 C_4_H_3_N_3_O_4_. 4 C_1_H_3_N_1_O_1_	2 C_4_H_3_N_3_O_4_. 2C_2_H_6_O_1_. H_2_O	C_4_H_3_N_3_O_4_. C_5_H_7_N_2_	2 C_4_H_3_N_3_O_4_. 2C_6_H_12_N_2_. 5H_2_O
Compound/ solvent	dioxane	piperazine	N,N'-dimethyl piperazine	pyridine	formamide	ethanol + water	3-amino pyridine	diaza bicyclo [2.2.2] octane + water
Formula wt	201.15	243.23	214.19	236.19	494.36	424.34	252.22	628.62
Crystal system	Monoclinic	Triclinic	Triclinic	Monoclinic	Monoclinic	Monoclinic	Monoclinic	Monoclinic
Space group	P 2(1)/n	P -1	P -1	P 2(1)/c	P 2(1)/c	P 2(1)/c	P 2(1)/c	P 2(1)/c
a (Å)	8.3535(12)	4.3579(5)	7.1052(14)	7.5173(4)	14.1865(8)	11.8264(8)	8.2825(7)	9.2323(10)
b (Å)	6.4277(10)	9.8014(12)	7.6010(20)	12.7817(8)	11.5840(7)	11.8738(8)	4.9546(4)	9.6598(10)
c (Å)	15.7030(20)	12.7310(20)	10.1500(20)	10.8938(7)	12.7907(7)	12.6511(8)	25.6080(20)	32.2899(6)
α (deg)	90	94.712(2)	93.023(3)	90	90	90	90	90
β (deg)	101.810(3)	99.452(2)	106.631(4)	97.780(5)	94.775(1)	98.696(1)	98.293(1)	90.801(1)
γ (deg)	90	97.603 (2)	117.689 (2)	90	90	90	90	90
Z	4	2	2	4	4	4	4	4
V(Å^3^)	825.30(2)	528.680(11)	453.8(2)	1037.080(11)	2094.70(2)	1756.10(2)	1039.870(15)	2879.40(7)
D_calc_ (Mg/m^3^)	1.619	1.528	1.567	1.513	1.568	1.605	1.611	1.45
R_1_	0.0688	0.0636	0.1409	0.0474	0.0439	0.0754	0.1235	0.0584
wR_2_	0.1561	0.1674	0.3522	0.1315	0.1244	0.2280	0.3196	0.1680
Goof	1.170	1.152	1.054	1.123	1.205	1.103	0.974	1.049
CCDC No.	267375	267376	267377	267378	267380	267381	267382	266627

## Results and discussion

**Scheme 1:**
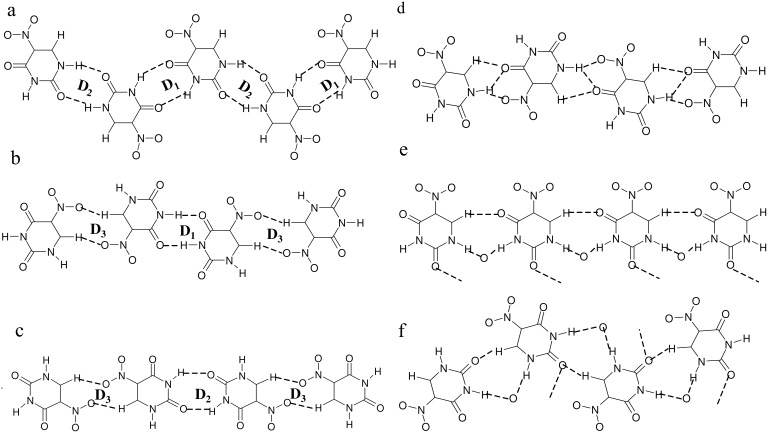
Hydrogen bonding patterns of **I** commonly found in its cocrystals with other molecules. Cyclic N-H...O (D_1_ and D_2_) and C-H...O (D_3_) dimeric bonds are marked.

In order to understand hydrogen bonded patterns in the cocrystals of **I**, it would be instructive to examine interactions that **I** itself can have in such cocrystals. **I** is nearly symmetric and has several hydrogen bond donor and acceptor sites, which provide a number of possibilities for hydrogen bonding. While the two-ring carbonyl oxygens and nitro-oxygens act as acceptors, the two NH groups along with the CH groups are donors. We present six hydrogen bonding possibilities in Scheme 1. Scheme 1a, where **I** involves two N-H...O cyclic bonds (R_2_^2^(8)) on either side, is similar to that found in centric nitrouracil structure. [21–22] We designate the two dimeric bonds as D_1_ and D_2_, based on the participating carbonyl oxygens. Thus, D_1_ and D_2_ are seen alternating along the molecular tape. Neighboring tapes interact through C-H...O interactions from the nitro-oxygens. A slight variation to this scheme is one where only one cyclic dimer, either D_1_ or D_2_ is present (Scheme 1b and Scheme 1c, respectively), alternating with cyclic C-H...O bonds (D_3_). The other structure in the contains bifurcated hydrogen bonds involving NH and CO groups of neighboring molecules forming a zig-zag arrangement (Scheme 1d). Another case is one where C-H...O and N-H...O interactions form a cyclicarrangement, R_3_^2^(9), involving the acceptor sites of the solvent molecules present in the cocrystal (Scheme 1e and Scheme 1f). The two schemes differ slightly in that e is linear while f appears staggered.

**Figure 1:**
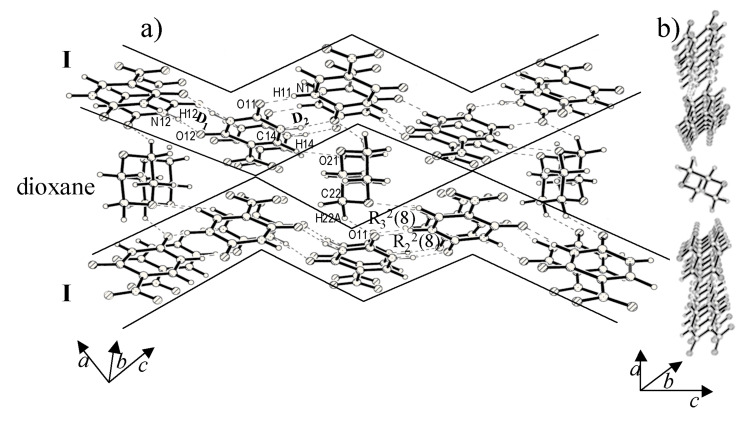
Dioxane molecules held between the zig-zag rows of **I**, viewed (a) perpendicular and (b) parallel to the molecular layer. Cyclic arrangements of bonds are marked using Etter's notation [11].

In the cocrystal of **I** and dioxane (with a 2:1 molar composition), the hydrogen-bonded tapes of **I** are retained (Figure 1) as in the parent structure. [21–22] Cyclic N-H...O dimers of both types, D_1_ and D_2_ are present with H...O distances of 1.79(3) and 1.84(4) Å respectively. This structure appears to belong to Scheme 1a. The dioxane molecule is accommodated between zig-zag chains of nitrouracil molecules giving rise to new C-H...O interactions in a R_3_^2^(8) arrangement (see Figure 1a). It is as though the neighboring rows of nitrouracil molecules slide against each other by approximately the size of a molecule thereby creating a cavity for the guest molecule. The molecular layers appear as a set of parallel planes as shown in Figure 1b.

**Figure 2:**
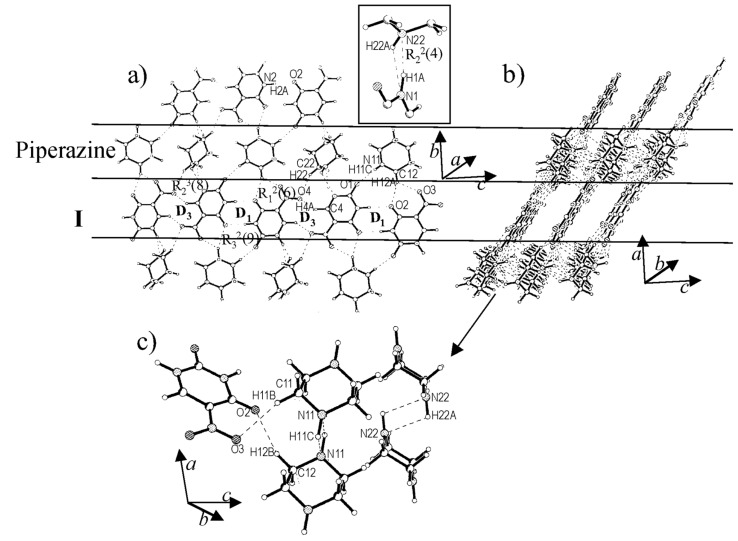
Cocrystal of **I** and piperazine: (a) A molecular layer showing alternating tapes of **I** and piperazine. Inset shows a cyclic N-H...O dimer formed between adjacent rows. (b) View along *b*-axis, showing layered structure and (c) Hydrogen bonds connecting adjacent layers.

Unlike the case of dioxane, cocrystallization of **I** with piperazine (1:1), destroys the hydrogen-bonded tape structure involving D_1_ and D_2_, and instead gives rise to a new structure with alternating rows of **I** and piperazine (Figure 2). Within the tape formed by **I**, only D_1_ type of N-H...O dimeric bonds are seen in addition to C-H...O type of interactions (H(4A)...O(4), 2.384(5) Å) involving the nitro groups. This situation is similar to the one shown in Scheme 1b where D_1_ and D_3_ dimeric bonds alternate along the molecular tape. The tapes of **I** are held in place by the hydrogen bonds from the neighboring rows of piperazine molecules, which do not interact among themselves (Figure 2a). There are two sets of C-H...O (H(22C)...O4, 2.540(5) Å) and dimeric N-H...N (H(1A)...N(22), 2.046(4) Å and H(22A)...N(1), 2.487(4) Å) bonds (see inset of Figure 2a) originating from the piperazine molecules that appear rotated in the given perspective. Alternating molecules make linear N-H...O contacts (H(11C)...O(1), 2.338(4) Å) in addition to bifurcated C-H...O contacts (H(12A)...O(3), 2.446(5) and H(12A)...O(2), 2.453(5) Å) involving acceptor oxygens from **I**. The **I**-piperazine layers are connected by dimeric N-H...N bonds as well as by C-H...O bonds, which are considerably weak due to unfavorable bond angles (~107°, see Figure 2c).

**Figure 3:**
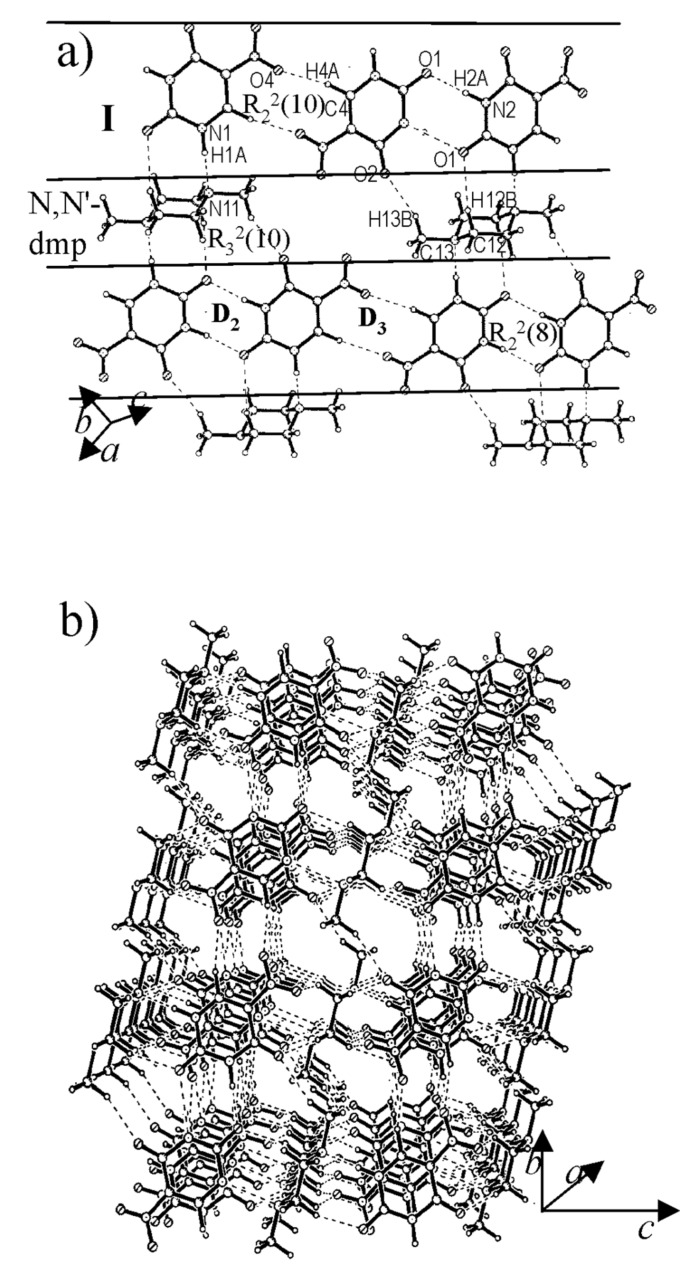
Cocrystal of **I** and N,N'-dimethylpiperazine: (a) A molecular layer showing alternating chains of **I** and N,N'-dimethylpiperazine, (b) A view along *a*-axis showing hydrophobic channels.

The cocrystal of **I** with N,N'-dimethylpiperazine (2:1), contains alternating rows of **I** and dimethylpiperazine (Figure 3), an architecture similar to that in the cocrystal of **I** with piperazine (see Figure 2). The main difference is that in the present case, the hydrogen-bonded tape of **I** is formed by D_2_ type of N-H...O dimers(H(2A)...O(1), 1.885(11) Å), alternating with the C-H...O dimers (D_3_). This bonding pattern may be referred to Scheme 1c. The central region of the dimethylpiperazine molecule makes mixed dimeric N-H...N, (H(1A)...N(11), 1.754(11) Å) and C-H...O (H(12B)...O(1), 2.504(13)Å) bonds with nitrouracils on either side. In addition, there exist C-H...O interactions from the methyl groups. The **I**-dimethylpiperazine layers are held by N-H...N and C-H...O bonds similar to the case in Figure 2b.

**Figure 4:**
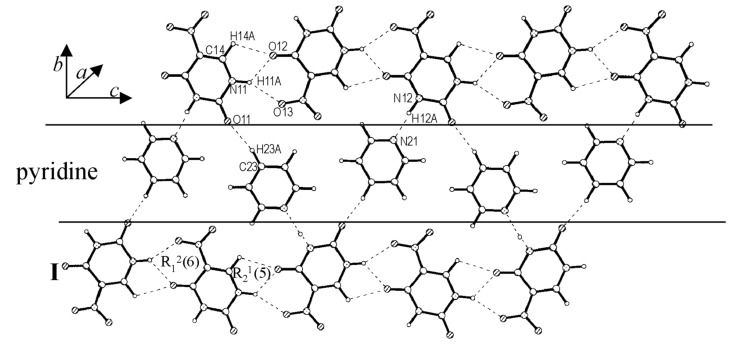
Cocrystal of **I** and pyridine: A molecular layer showing alternating rows of **I** and pyridine.

In the cocrystal of **I** with pyridine (1:1), there are no cyclic dimeric bonds although the alternating tape structure is retained (Figure 4). Thus, the tapes of **I** are stabilized by bifurcated N-H...O and C-H...O bonds as shown in Scheme 1d. The corresponding distances for N-H...O are (H(11A)...O(4) Å, 2.048(3) and H(11A)...O(13), 2.188(3) Å and for C-H...O,(H(14A)...O(12), 2.400(3) Å). The pyridine molecules make linear N-H...N, (H(12A)...N(21), 1.810(3) Å) and C-H...O, (H(23A)...O(11), 2.216(4) Å) bonds with the nitrouracil molecules. Pyridine being a strong electron donor, it is not surprising that the characteristic hydrogen-bonded dimeric structure present in the parent crystal (centric form of **I**, Ref. 21) is destroyed in this cocrystal.

Such hydrogen bond breaking ability of good electron donors becomes more evident when we examine the structure of the molecular tapes in the cocrystal of **I** with DMSO[21] (also see CCDC – 267379). In this case, the rows of **I** are stabilized by C-H...O contacts (H(4)...O(2), 2.488(10) Å) along the tape and bifurcated N-H...O bonds originating from the sulfoxide oxygens of DMSO giving rise to a cyclic arrangement, R_3_^2^(9), similar to Scheme 1e. The other signature of this bonding pattern is a C-H...O interaction (parallel to N-H...O), involving one of the carbonyl oxygens and a methyl hydrogen. In addition, the sulfur atom of the DMSO seems to interact through a N-H...S contact (H...S, 2.664(19) Å). The nitrouracil-DMSO layers arrange in a zig-zag manner with the methyl groups of DMSO sticking out in the interlayer region.

**Figure 5:**
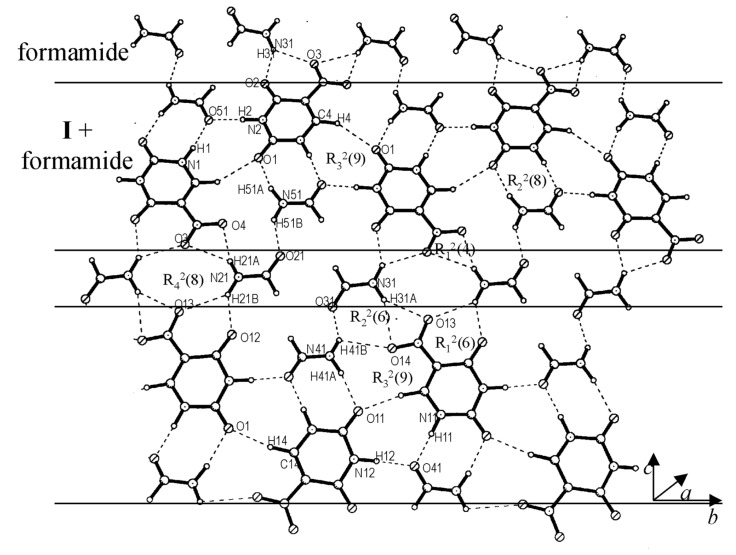
Cocrystal of **I** and formamide: A molecular layer showing tapes of mixed composition,**I** and formamide, alternating with formamide chains.

All the cocrystals discussed so far, have essentially a layered structure with alternating tapes of **I**, the guest molecule being stabilized by different modes of hydrogen-bonding (Scheme 1a to Scheme 1e). In the cocrystal of I with formamide (1:2), the layered structure is retained but the tapes have a mixed composition, comprising both **I** and formamide molecules in alternating positions enclosed on either side by rows of formamide molecules (Figure 5). The nitrouracil molecules in a tape are rotated with respect to their cousins by 120° and do not have any direct interaction except for a C-H...O contact (H(4)...O(1), 2.334(13) Å and H(14)...O(11), 2.222(11) Å). The nitrouracil molecule is bonded to formamide through a cyclic N-H...O dimer, (H...O, 1.71 – 2.12 Å), which resembles closely D_2_ in Scheme 1a. It is as though formamide mimics a portion of the nitrouracil molecule. This leads to a highly optimized hydrogen bond pattern with several possible cyclic arrangements, as marked in the figure and illustrated by Scheme 1f. From the crystal structure, it appears that both DMSO and formamide, which exhibit high dielectric constants can significantly influence the hydrogen bonding pattern in the cocrystal with **I**.

**Figure 6:**
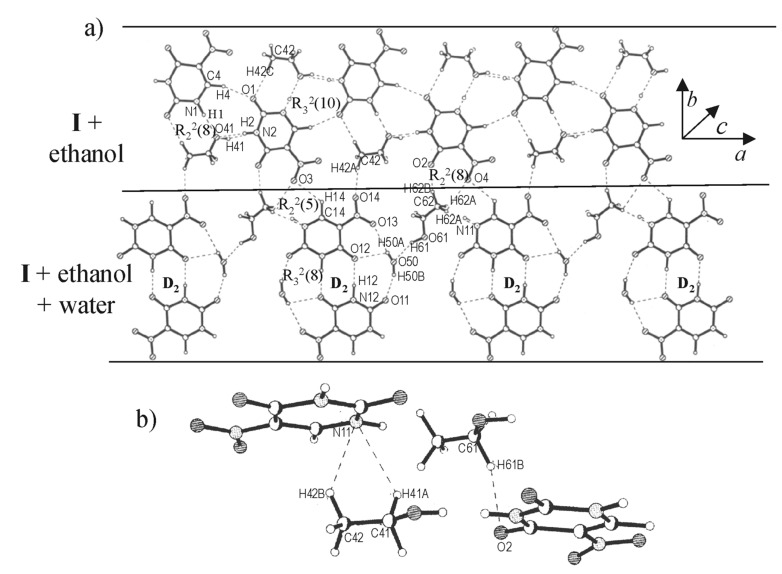
Cocrystal of **I**, ethanol and water: (a) A molecular layer showing tapes of mixed compositions- tapes containing **I** and ethanol alternating with those containing **I**, ethanol and water, (b) Hydrogen bonds connecting adjacent layers.

Crystallization of **I** from aqueous ethanol yielded a cocrystal incorporating water as well as ethanol. The molecular packing in the cocrystal of **I** with ethanol and water bears some resemblance to the structures described earlier. It forms a layered structure, with each layer containing alternating molecular tapes of **I**-ethanol and **I**-ethanol-water (Figure 6). The molecular arrangement in the **I**-ethanol tape is similar to that of **I**-formamide in Figure 5 (Scheme 1f). The bonding in the **I**-ethanol-water tape is, however, different. Here, pairs of nitrouracils molecules are bonded through dimeric N-H...O bonds (D_1_) besides four O-H...O bonds (H...O, 1.86 – 2.32 Å) induced by two water molecules from strategic positions, thus forming a set of parallel-antiparallel bonding arrangement. A similar bonding pattern has been observed for a monohydrate of 5-nitrouracil. [27] It resembles the quadruple hydrogen bond pattern found usually in copolymer and peptide structures. [28–30] The two tape structures alternate in a layer with C-H...O bonds from the methyl group connecting them. Interlayer contacts (see Figure 6b) are of the type, C-H...O and C-H...N (H(61B)...O(2), 2.324(6) Å; H(42B)...N(11), 2.614(6) Å and H(41A)...N(11), 2.353(6) Å).

**Figure 7:**
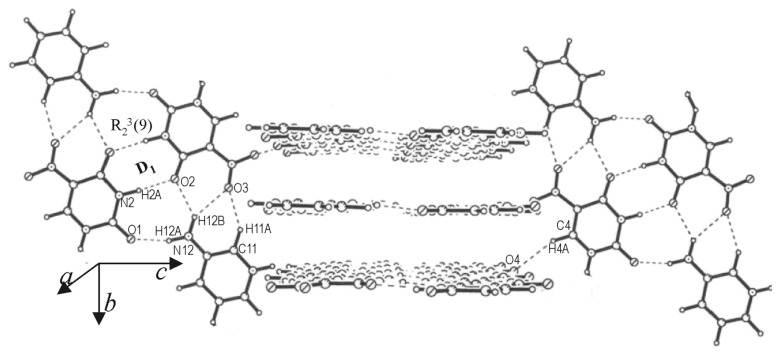
Cocrystal of **I** and 3-aminopyridine: Stacks of molecular tapes arranged in alternating parallel and perpendicular orientations.

Figure 7 shows the packing in the cocrystal of **I** with 3-aminopyridine, where we readily identify the bonding motif of Figure 6, albeit with a difference. Due to the presence of the amino group from aminopyridine, the bonds belonging to the parallel-antiparallel arrangement are of N-H...O type (H(2A)...O(2), 1.919(7) Å and H(12A)...O(1) 1.908(7) Å), involving the D_1_ motif. We also notice that bifurcated N-H...O and C-H...O bonds connect adjacent **I** and aminopyridine, much like in the **I**-pyridine cocrystal (Figure 4 and Scheme 1d). Unlike the cocrystal structures discussed so far, the molecular dimers in this structure do not extend sidewise into infinite layers but instead allow similar motif to stack perpendicularly as depicted in the figure. The two systems are bonded though several C-H...O and C-H...N interactions.

**Figure 8:**
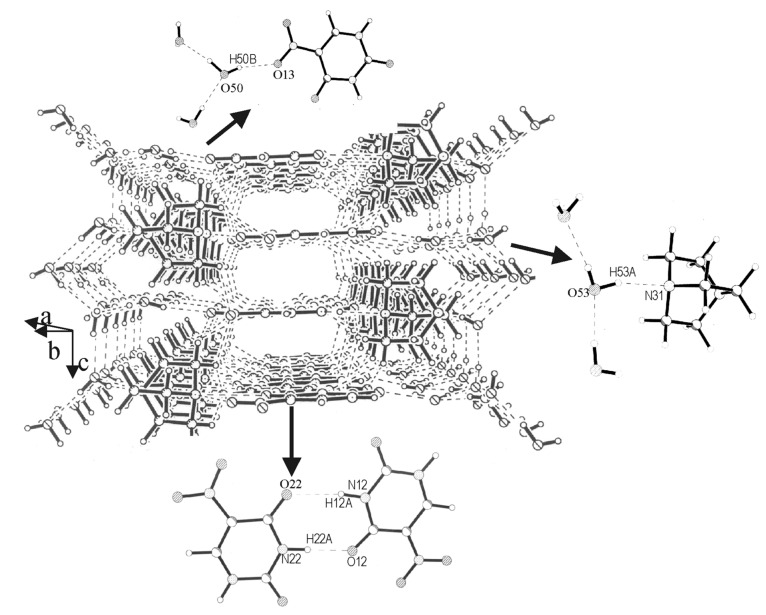
Cocrystal of **I** and DABCO: A view along the diagonal of the *ab* plane showing the supramolecular assembly. Inset shows some important hydrogen bonding patterns.

Another example of such stacking has been found in the cocrystal of **I** with DABCO and water (molar composition, 2:2:5), as shown in Figure 8. The structure contains cyclic dimers of nitrouracil (see inset of Figure 8) formed through N-H...O interactions (H(22A)...O(12), 1.878(3) Å and H(12A)...O(22), 2.189(3) Å), arranged in the form of parallel set of tapes. The nitrouracil tapes are held between sheets of the DABCO molecules, which in turn are covered by layers of water molecules. These supramolecular assemblies get rotated along the *a* axis by 90° alternately. There is extensive hydrogen bonding between DABCO and nitrouracil (N(11)-H(11A)...N(32), 1.940(4) Å; C-H...N, 2.43 – 2.52 Å and, C-H...O, 2.45 – 2.57 Å), water and DABCO (O-H...N, 1.98(6) Å) as well as between water and nitrouracil (O-H...O, 2.06(6) and 2.11(5) Å). The donor-H-acceptor angles are generally favorable, N-H...N (153°, O-H...N (162° – 163°), O-H...O (161° – 178°) and C-H...N (145°). There are also some C-H...O interactions, with the angle in the 118° – 152° range.

**Figure 9:**
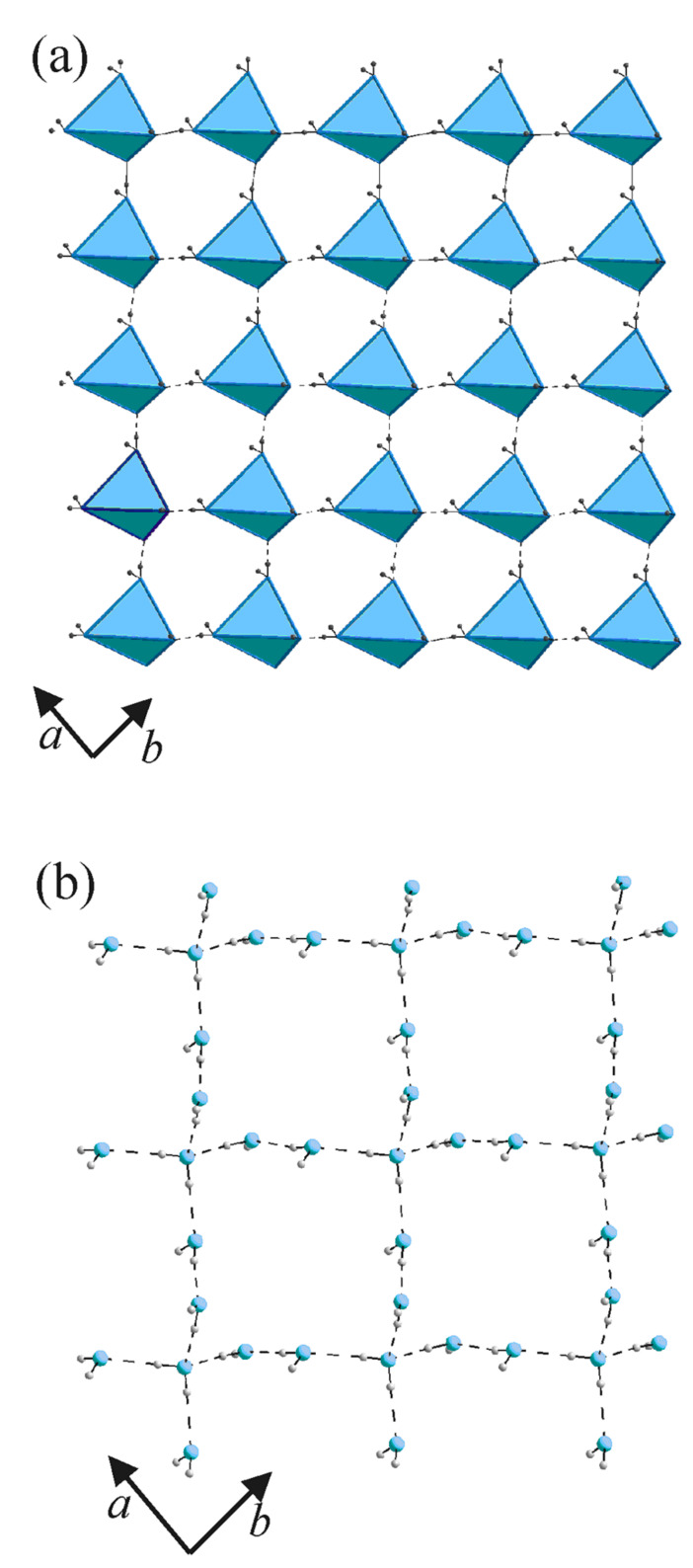
(a) Tetrahedral pentamers of water in the *ab* plane forming a nearly square arrangement (b) Hydrogen bonded network in the water layer.

**Figure 10:**
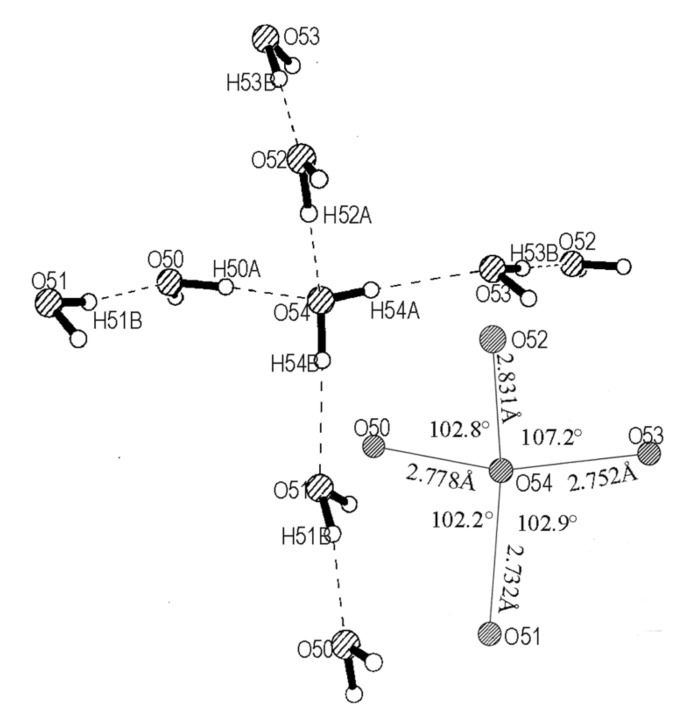
A tetrahedron pentamer of water. The O...O distances and H-O-H angles are indicated in the inset.

The significant aspect of this supramolecular system is the structure of the water layer, consisting of a well-defined, isolated network of hydrogen-bonded acyclic pentamers as shown in Figure 9. This is yet another example of a supramolecular assembly hosting acyclic pentamer clusters of water. [31–36] The tetrahedral pentameric unit corresponds exactly to that described by Walrafen,[37–38] involving a central H_2_O molecule where the two hydrogen donors of the central molecule engage two water molecules, while the two lone-pairs of the oxygen act as acceptors bringing in two more water molecules (Figure 10). The O...O distances are in the 2.73 – 2.83 Å range, comparable to the values obtained by *abinitio* calculations on water oligomers. [39–40] Three of the H-O-H angles involving the central molecule are within 0.4° of the average value of 102.6°,(see inset of Figure 10) while the other H-O-H angle, extended from the outer proton donors, is 107.2° due to lone-pair lone-pair repulsion. Besides the hydrogen bonds with the central molecule, the hydrogens of the molecules at the tetrahedral corners bond alternatively to DABCO and nitrouracil molecules above and below the water layer. It appears as though the tetrahedral pentamers are formed to stabilize the water molecules bonded to DABCO and nitrouracil molecules.

The tetrahedral pentamers in the water layer are arranged in a nearly square lattice in the *ab* plane, with basis vectors of 6.58 and 6.78 Å, rotated with respect to the parent lattice by ~45° (Figure 9). The tetrahedral units are connected to one another through a O-H...O bonds with O...O distances of 2.83 and 2.85 Å in the two directions, which are slightly longer than those found inside the tetrahedra (see Figure 10). The array of water molecules in Figure 9b can be described as a network of cyclic dodecameric species sharing the four edges.

## Conclusion

The present study of the cocrystals of 5-nitrouracil, **I**, brings out clearly the high propensity of the molecule in forming interesting hydrogen bond patterns. Of the several cocrystals investigated by us, all except those involving 3-aminopyridine exhibit layered structures with layers comprising two different compositions. In the cocrystals of **I** with piperazine, N,N'-dimethylpiperazine, diazabicyclo [2.2.2]octane, pyridine and DMSO, tapes of **I** alternating with rows of the partner molecule occur, while in other cocrystals with formamide and ethanol, the molecular tapes exhibit mixed compositions. In the cocrystal of **I** with dioxane, the hydrogen bonding pattern of **I** is somewhat modified compared to the parent centric structure, where instead of tapes, dimers of **I** are present. Here, cyclic N-H...O dimeric bonds (alternating D_1_ and D_2_) are seen on either side of the molecule (Scheme 1a). In some cases, dimeric C-H...O bonds (D_3_) are seen alternating with D_1_ (**I**- piperazine) or D_2_ (**I**- N,N'-dimethylpiperazine) as in Scheme 1b and Scheme 1c. A new type of bonding pattern is seen in the cocrystal of **I** with pyridine involving zig-zag arrangement of a linear C-H...O and bifurcated N-H...O bonds (Scheme 1d). Cocrystals with DMSO and formamide exhibit tapes of **I** where bonding between adjacent nitrouracil molecules involves an acceptor oxygen from the partner molecule (Scheme 1e and Scheme 1f). Another interesting feature of these cocrystals is the presence of a quadruple-like hydrogen bonding pattern involving either O-H...O (**I**-ethanol-water) or N-H...O (**I**-3-aminopyridine)bonds. The presence of a network of acyclic tetrahedral pentamers of water in the cocrystal of **I** with diazabicyclo [2.2.2]octane is also a novel finding.
